# Rethinking population health in practice: pediatric immunizations as a policy and system integration case

**DOI:** 10.3389/fpubh.2026.1749357

**Published:** 2026-03-18

**Authors:** Diego R. Hijano

**Affiliations:** 1 Department of Infectious Diseases, St. Jude Children’s Research Hospital, Memphis, TN, United States; 2 Department of Pediatrics, University of Tennessee Health Science Center, Memphis, TN, United States

**Keywords:** population health, pediatric immunization, implementation science, health equity, vaccine policy, global health systems

## Abstract

This conceptual policy analysis examines the persistent gap between population health frameworks and their implementation in practice, using pediatric immunizations as a case example. Declining vaccine coverage and widening geographic disparities illustrate how broad definitions of population health may lack the operational structure needed to guide coordinated governance and delivery. Informed by a structured narrative review of peer-reviewed literature, policy reports, and national surveillance data, the Rainbow Model of Integrated Care is applied to analyze how clinical, professional, organizational, system, functional, and normative domains shape immunization performance across preventive care systems. The analysis identifies policy-relevant levers, including interoperable data infrastructure, alignment of exemption governance, integration of equity-focused payment incentives, community-partnered outreach, and transparent performance monitoring. For each domain, implementation considerations, authority structures, privacy safeguards, resourcing implications, and potential political and ethical trade-offs are examined. Illustrative measurable indicators are proposed to operationalize integration and support accountability. Although grounded primarily in the United States context, the challenges described and structural considerations discussed are relevant to other decentralized health systems. This analysis suggests that structured integration frameworks may assist policymakers in translating population health principles into coordinated and ethically grounded implementation strategies for immunization systems.

## 1 Introduction

The United States is witnessing a resurgence of measles, a disease previously declared eliminated, with reported cases increasing markedly in recent years following prolonged periods of low transmission (1, 2). At the same time, national kindergarten vaccination coverage has declined from approximately 95% in the late 2010s to below 93% in recent school years, coinciding with a continued rise in non-medical exemptions (3, 4). These trends, occurring in a context marked by vaccine skepticism and political polarization, reflect a widening disconnect between public health goals and system performance. They also highlight broader difficulties in translating population health concepts into coordinated policy and practice.

Operationalizing the population health framework is both possible and necessary for strengthening preventive care systems. Pediatric immunizations offer a clear and measurable public health intervention that can bridge the divide between theory and implementation. When supported by coordinated electronic health records, school based services, public and private partnerships, culturally responsive communication, and aligned state and federal policies, vaccination performance becomes a sensitive indicator of system integration and equity (5).

This article reexamines the relevance and practical utility of the population health framework in the current United States policy environment. It argues that the broad conceptual scope of the framework, while theoretically inclusive, often limits its operational usefulness. Using the Rainbow Model of Integrated Care as an organizing structure, this analysis examines how integration across clinical, professional, organizational, system, functional, and normative domains can strengthen coherence, accountability, and equity. Grounded in the case of pediatric immunizations, the analysis identifies points where fragmentation occurs and outlines actionable strategies to narrow the gap between intent and impact.

### 1.1 Approach and evidence sources

This manuscript is a conceptual policy analysis informed by a structured narrative review of peer-reviewed literature, policy reports, and national surveillance data. The goal was not to conduct a systematic review or formal evidence grading, but to synthesize policy-relevant evidence to examine how integration frameworks can support translation of population health principles into operational strategy.

Relevant sources were identified through iterative review of major medical and health policy journals, reference lists of foundational articles, and publicly available reports from national and international public health agencies, including the Centers for Disease Control and Prevention (CDC), the World Health Organization (WHO), and the Organisation for Economic Co-operation and Development (OECD). The primary time frame emphasized publications from 2000 to 2025, with particular attention to evidence published since 2015 addressing pediatric immunization systems, exemption policy, immunization information systems, governance mechanisms, and health equity implementation.

Searches were conducted using academic databases and policy repositories, including PubMed/MEDLINE, Scopus, Google Scholar, and official agency portals (e.g., CDC, WHO, OECD). Sources were screened for relevance to pediatric immunization delivery, governance structures, and integration frameworks based on title and abstract review, followed by full-text assessment when appropriate. Priority was given to nationally representative surveillance data, widely cited empirical studies, and policy analyses directly addressing immunization systems and implementation.

Sources were selected for inclusion if they (1) examined immunization delivery systems or policy design; (2) addressed integration, governance, or accountability structures relevant to population health; or (3) provided national or multi-jurisdictional surveillance data on vaccination coverage, exemption trends, or outbreak patterns. When evidence was inconsistent, priority was given to national surveillance data, systematic reviews, and multi-state analyses. Observational findings were interpreted cautiously, and causal language has been avoided where evidence remains correlational.

**Figure 1:**
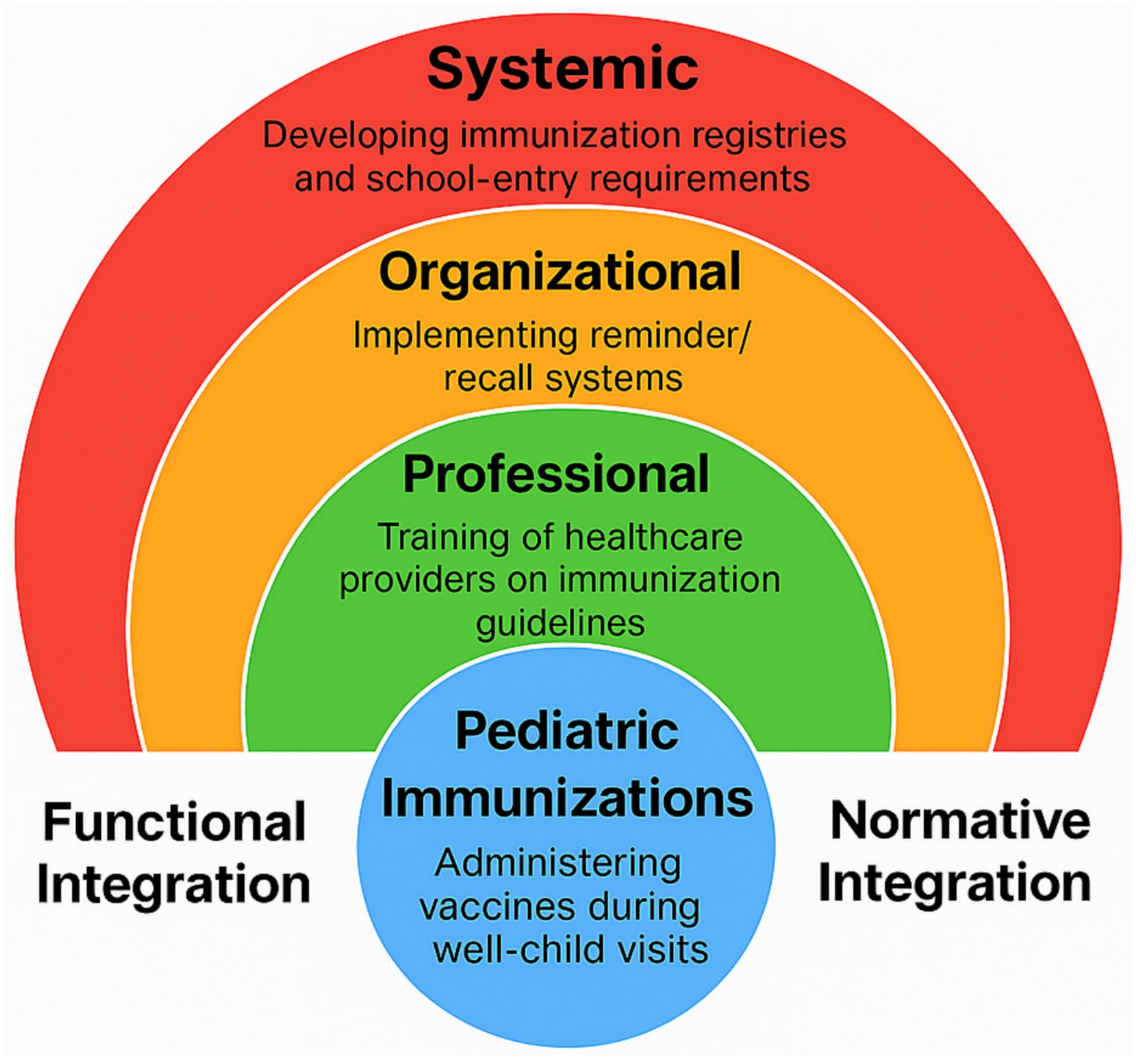
Rainbow model of integrated care applied to pediatric immunizations. This author-created figure illustrates the six domains of the Rainbow Model of Integrated Care (10, 11) as applied analytically to pediatric immunization delivery. Clinical, professional, organizational, system, functional, and normative integration are represented as interrelated layers that shape how services are aligned across care settings and communities. Applied to pediatric immunization programs, the model illustrates how fragmentation at any layer may contribute to disparities in coverage and how coordinated improvements across domains can strengthen equitable and population-focused implementation.

This approach enables structured synthesis of implementation-relevant evidence while acknowledging that selection was non-systematic and may not capture all published interventions. The analysis is intended to clarify policy design, integration, and governance considerations rather than to provide a comprehensive empirical review of all immunization strategies (Figure 1).

## 2 Population health as a policy framework: conceptual tensions and practical limitations

The population health framework, classically defined as “the health outcomes of a group of individuals, including the distribution of such outcomes within the group,” was originally intended to bridge clinical care, public health, and policy (6). By emphasizing outcomes and their distribution, it positioned equity as central to health improvement. At the same time, the World Health Organization’s Health for All agenda advanced the idea that every person is entitled to the highest attainable standard of health (7). Together, these perspectives emphasized cross sector collaboration consistent with evolving determinants-of-health frameworks in public health scholarship (8).

Despite its conceptual strength, the broad scope of the population health framework often limits its operational usefulness in policy and delivery settings (9). Over the past two decades, the term has undergone considerable conceptual drift (Table 1). Health systems, insurers, and policymakers have increasingly applied it to activities such as patient segmentation, risk stratification, and cost containment rather than broader efforts to reduce inequality, improve education, or address structural conditions that shape health (10). Its overlap with related terms, including public health, community health, and health promotion, has further blurred boundaries and contributed to fragmented accountability across sectors (Table 2).

**Table 1:** Selected definitions of population health.

Source/Organization	Definition summary
Kindig and Stoddart (6)	Defines population health as “the health outcomes of a group of individuals, including the distribution of those outcomes,” emphasizing measurement, equity, and the connection between policy and health (6).
World Health Organization (34)	Conceptualizes health as a state of complete physical, mental, and social well-being, providing the foundational principle for later population health frameworks (34).
Centers for Medicare and Medicaid Services (CMS)	Frames population health in terms of group level outcomes shaped by contextual factors, often linked to accountable care, quality improvement, and coverage strategies (35).
Centers for Disease Control and Prevention (CDC)	Describes population health as an adaptable approach that aligns practice and policy to improve outcomes for defined groups (36).
National Academy of Medicine (2002)	Calls for an intersectoral public health system capable of addressing upstream determinants such as housing, education, and income (37).
American Public Health Association (APHA)	Emphasizes health outcomes, equity, and social justice, centering the structural determinants that shape population health (38).
Interdisciplinary Association for Population Health Science (IAPHS)	Promotes a multilevel framework that links biological, social, and policy factors, with particular attention to place based disparities (39).
New York State Department of Health (NYSDOH)	Defines population health as group focused and distinct from individual care, emphasizing collective outcomes and the role of policy level interventions (40).

**Table 2:** Comparison of Related Health Concepts.

Domain	Definition	Primary focus	Scope	Key actors	Example: pediatric immunizations
Population health	The health outcomes of a group of individuals and the distribution of those outcomes within the group	Outcomes, determinants, equity	Geographic, demographic, systemic	Health systems, researchers, policymakers	Assessing neighborhood level immunization disparities to guide targeted outreach and reduce inequities
Public health	Collective action to protect and promote the health of populations and prevent disease	Protection, promotion, prevention	Local, regional, national	Government agencies, public health departments	Implementing school entry immunization requirements and coordinating publicly funded vaccine programs
Community health	The health status, needs, and priorities of a defined community, often shaped by place, culture, or shared identity	Local disparities, engagement	Neighborhoods, municipalities, community networks	Local health departments, coalitions, community based organizations	Partnering with community leaders to provide culturally grounded vaccine education and access in trusted settings
Health promotion	A process that enables individuals and communities to increase control over their health and improve wellbeing	Education, empowerment, behavior change	Individual to population	Educators, nongovernmental organizations, clinicians, media partners	Developing communication campaigns through schools, clinics, and community platforms to build confidence in routine vaccines

Although initiatives such as the National Academy of Medicine’s reports and state level efforts have sought to refocus attention on upstream factors, adoption has been inconsistent. The framework still lacks an organizing structure that connects broad intent to operational execution. Policymakers and health systems frequently endorse its goals but struggle to translate them into coordinated action aligned with equity. The Rainbow Model of Integrated Care (RMIC), originally developed to conceptualize integration across healthcare systems, offers such a structure (11–13). The model delineates six interrelated domains—clinical, professional, organizational, system, functional, and normative—that together describe how services, governance, and shared values are aligned across levels of care. It has been applied internationally to examine how coordinated delivery systems can better connect service organization with population-level outcomes (12–14). For population health, it provides the specificity needed to translate high-level goals into multisector strategies that embed coordination and equity from design through implementation.

## 3 Pediatric immunizations in the United States: a case of fragmented implementation

Pediatric immunizations illustrate how a population health framework can struggle to guide practice when it lacks operational structure. In the United States, vaccine mandates and delivery strategies vary widely across states, producing significant disparities in coverage, exemption policies, and system support.

States such as Mississippi and West Virginia, which allow only medically justified exemptions, consistently report some of the highest vaccination rates in the country. In contrast, states with more permissive exemption policies, including Texas, Idaho, and Oregon, report lower coverage and have experienced recurrent measles outbreaks, patterns that are associated with policy variation and broader contextual factors (3, 4). These patterns reflect not only differences in public trust and local norms but also divergent policy decisions, financing mechanisms, and governance structures.

Variation also extends to immunization information systems. Some jurisdictions maintain robust registries with timely reporting, while others rely on delayed or incomplete data, limiting their ability to identify children who are overdue for vaccines or respond rapidly to local outbreaks (15, 16). Incompatible technologies and inconsistent data sharing agreements further hinder coordination among providers, schools, and public health agencies.

Taken together, these gaps demonstrate how fragmented governance and system design limit the ability of a population health framework to drive coherent action. In the absence of common metrics, shared accountability, and aligned policies, efforts to improve childhood vaccination remain uneven. Applying the Rainbow Model of Integrated Care to this case reveals where coordination breaks down and helps identify opportunities for stronger alignment that could enhance equity and strengthen immunization performance.

## 4 Application of the rainbow model to immunization delivery

The Rainbow Model of Integrated Care provides a structured framework for analyzing how coordination across multiple sectors can strengthen population health implementation (11–14). Applied to pediatric immunizations in the United States, each of its six domains highlights specific gaps and opportunities to improve alignment, equity, and system performance.

### 4.1 Clinical integration

Clinical integration focuses on coordinating preventive services across the full care continuum. For immunization delivery, this requires embedding vaccine readiness and follow up into every step of routine pediatric care. Before the visit, reviewing immunization records helps clinics identify children who are due for vaccines. Staff can use previsit planning tools within electronic health records to flag gaps and notify clinicians, while automated reminders such as text messages or phone calls prepare families and reduce missed opportunities.

During the encounter, care teams reinforce a consistent message that vaccines are a routine and expected part of child wellness. Staff confirm immunization status, nurses prepare doses, and clinicians use electronic prompts to ensure all recommended vaccines are offered. After the visit, scheduling booster doses and recording completions in immunization registries maintain continuity. Feedback loops allow providers to track progress and target children who fall behind. These integrated workflows position vaccination as a core feature of comprehensive pediatric care rather than a supplemental activity.

### 4.2 Professional integration

Professional integration emphasizes collaboration and shared accountability among the diverse professionals involved in vaccine delivery. Pediatricians, school nurses, pharmacists, and public health staff often work in parallel with limited coordination. Although guidance from national organizations provides direction, differences in emphasis or scope sometimes lead to inconsistent practice.

Greater alignment is possible through joint initiatives such as interdisciplinary training, shared quality improvement collaboratives, and professional statements defining referral pathways and documentation standards. In areas where access to providers is limited, collaborative practice agreements between pharmacists, nurses, and clinics support continuity. When professionals view immunization as a shared responsibility rather than a siloed task, coordination strengthens and access becomes more equitable across settings.

### 4.3 Organizational integration

Organizational integration involves aligning the institutions responsible for immunization delivery. Fragmented relationships among health departments, schools, clinics, and community organizations often result in uneven coverage. These challenges are intensified in jurisdictions where public health resources are limited or where school-based vaccination encounters political or parental resistance.

Stronger collaboration requires joint planning, shared resources, and visible leadership across sectors. Health departments can coordinate schools, pediatric practices, hospitals, and community organizations around common immunization goals such as back to school campaigns or catch-up efforts. When institutions work together, families receive consistent messages and services become easier to access. By clarifying roles and strengthening shared accountability, organizational integration transforms immunization from isolated programs into a coordinated community effort.

### 4.4 System integration

System integration concerns the alignment of policies, regulations, and financing mechanisms that shape vaccine delivery. Variation in mandates, exemption policies, and reimbursement structures across jurisdictions creates inconsistent incentives and uneven coverage. When these structures are misaligned, even strong clinical and organizational efforts struggle to achieve sustained improvement.

Effective system integration requires policy and financial alignment across levels of government. National or state authorities can establish minimum standards for immunization policy, surveillance, and financing while allowing flexibility in implementation. Baseline protections, including school entry requirements and transparent reporting, should be consistent across regions. Reimbursement mechanisms must also support outreach and follow up, particularly in underserved areas. Linking funding to performance in high-need communities encourages shared accountability. When systems treat vaccination as a population level investment, coordination and equity are strengthened across the RMIC domains.

### 4.5 Functional integration

Functional integration refers to the technical and administrative infrastructure that enables coordination. Immunization delivery depends on systems that connect clinical practice, public health agencies, and schools. High performing regions employ interoperable registries that allow timely exchange of vaccine data, identify children who are overdue, and support population level monitoring. In many jurisdictions, however, reporting is delayed or incomplete, limiting the ability to respond to gaps in real time.

Improving functional integration requires governance, standardization, and sustained investment. Shared data standards, unique patient identifiers, and secure exchange protocols help maintain continuity across settings. Integrating immunization data into electronic health records strengthens clinical decision making and quality improvement. Investment in public health informatics is an equity strategy because regions with weaker infrastructure fall further behind. Functional integration ensures that every child is visible in the data and included in planning efforts.

### 4.6 Normative integration

Normative integration encompasses the shared values, trust, and relationships that support engagement across the health system. In an environment shaped by vaccine skepticism and polarized information, this domain is essential yet fragile. Trust involves confidence in science, institutions, and interpersonal relationships, and erosion in any area can undermine vaccine uptake.

Building normative integration begins with listening and empathy. Concerns about vaccines often arise from fear, misinformation, or previous negative encounters with healthcare. Behavioral frameworks such as EAST (Easy, Attractive, Social, Timely) (17) and MINDSPACE (Messenger, Incentives, Norms, Defaults, Salience, Priming, Affect, Commitments, Ego) (18) show that vaccine decisions are influenced by social norms, credible messengers, and emotional context rather than information alone. Addressing structural barriers enhances the reach and impact of these strategies (19, 20).

Strengthening confidence requires trusted messengers, culturally responsive communication, and repeated, respectful interactions. Countering misinformation calls for humility and sustained engagement rather than confrontation. When people feel heard and understood, they are more willing to act on public health guidance. Normative integration shifts vaccination from a transactional action to a relationship centered process supported by shared purpose.

Taken together, the six domains of the Rainbow Model illustrate where fragmentation limits immunization performance and where targeted policy and practice changes can strengthen coordination, equity, and system impact. These insights form the basis for the recommendations that follow.

## 5 Policy and practice recommendations

Translating the population health framework into practice requires deliberate alignment across public health, clinical care, and policy systems. Pediatric immunizations illustrate where fragmentation produces inequities and inefficiencies. The recommendations below, grounded in the domains of the Rainbow Model of Integrated Care, address structural gaps in immunization strategy and outline pathways toward sustainable and equitable implementation.

### 5.1 Establish national standards for vaccine data interoperability

A critical first step is the development of national immunization data standards that allow vaccine information to move securely across regions and care settings. In many countries, including the United States, immunization registries operate at the state or local level, resulting in fragmented and incomplete data.

Nationally coordinated interoperability standards would enable information to flow between electronic health record systems, public health departments, and schools in real time. Providers could access verified records through standardized identifiers, and families and schools could retrieve information when needed. Achieving this requires sustained investment in digital infrastructure, strong governance to protect privacy, and alignment with national quality frameworks.

By linking registry data to pediatric outcome measures and establishing clear accountability, interoperable systems can strengthen coordination, reduce inequities, and improve vaccination coverage.

#### 5.1.1 Implementation and governance considerations

Authority for immunization information systems in the United States primarily resides at the state level, while federal agencies such as the Centers for Disease Control and Prevention (CDC) and the Office of the National Coordinator for Health Information Technology (ONC) can establish interoperability standards and provide financial incentives (12, 13). Implementation would therefore require a federated model: national technical standards coupled with state-level execution.

Legal and privacy constraints include compliance with HIPAA, FERPA, and state-specific data protection statutes. Cross-sector exchange involving schools and public health agencies must clearly define permissible use, parental notification requirements, and safeguards against secondary data misuse. Prior analyses of immunization information systems emphasize that data governance clarity and public transparency are central to maintaining trust and sustaining participation (12, 13).

Resourcing implications include sustained federal and state investment in public health informatics infrastructure, workforce training, and cybersecurity protections. Systematic reviews of immunization information system implementation note that under-resourced jurisdictions face persistent interoperability gaps, contributing to inequitable visibility of children who are overdue for vaccines (13).

Sequencing would likely involve (1) establishing minimum technical standards aligned with national health information exchange frameworks, (2) aligning federal grant funding with compliance benchmarks, (3) piloting interstate exchange models, and (4) expanding integration nationally. Incremental implementation reduces disruption and allows evaluation of technical and operational barriers before broader rollout.

Political and operational risks include resistance to perceived federal overreach and concerns about centralized data systems. Mitigation strategies include preserving state control over registry governance, using conditional funding rather than mandates, and emphasizing that interoperability enhances local outbreak response capacity and equity-focused outreach (12).

### 5.2 Reform exemption policies through federal and state alignment

Reducing exemptions that are not medically justified requires coordinated governance that balances national standards with regional implementation. Although school vaccination mandates typically fall under state authority, national leadership can establish minimum requirements for public institutions and encourage alignment across private schools (4). States that restrict non-medical exemptions tend to report higher vaccination coverage, while those with broader exemption allowances often experience lower coverage and periodic outbreaks, although these patterns reflect multiple interacting factors including trust, political context, and enforcement consistency (3, 4). Policy alignment alone is therefore unlikely to be sufficient but remains an important structural lever within broader integration efforts.

National guidance could clarify minimum evidentiary standards for medical exemptions and encourage uniform documentation and verification processes (4). Federal or national agencies may also condition certain education or public health funding streams on compliance with evidence-informed immunization standards, while preserving state-level implementation flexibility.

Coordinated federal and state strategies can reduce geographic disparities, enhance transparency, and promote more consistent governance across jurisdictions.

#### 5.2.1 Implementation and governance considerations

In the United States, primary authority over school entry immunization requirements resides with state legislatures under established public health police powers. Federal agencies cannot directly mandate state exemption policy but may influence alignment through conditional funding mechanisms, regulatory guidance, and interstate coordination frameworks.

Legal constraints include constitutional considerations related to state authority, religious accommodation jurisprudence, and equal protection principles. Policy reform must therefore proceed through state legislative processes, supported by model statutory language, stakeholder engagement, and clear documentation standards. National professional organizations and public health associations can play a coordinating role in promoting evidence-based exemption criteria (4).

Resourcing implications include administrative capacity for exemption review, auditing systems to prevent misuse, and clinician education regarding appropriate medical contraindications. Sequencing would likely involve (1) standardizing exemption documentation requirements, (2) enhancing transparency through public reporting of exemption rates, (3) aligning funding incentives, and (4) evaluating effects before broader statutory reform.

Political risks are substantial. Exemption reform is often framed within debates about individual liberty and parental rights. Efforts perceived as coercive may intensify resistance or fuel misinformation. Mitigation strategies include transparent communication about the public health rationale, phased implementation, equity safeguards for medically vulnerable populations, and avoidance of punitive measures that disproportionately affect under-resourced communities.

Importantly, exemption policy reform should be evaluated alongside complementary interventions in clinical integration, data infrastructure, and community engagement. Structural alignment across RMIC domains increases the likelihood that policy changes translate into sustained improvements in coverage.

### 5.3 Promote integrated delivery models through payer and provider alignment

Equitable immunization coverage depends on financial and operational frameworks that support preventive care. Public payers such as Medicaid, and their regional equivalents, can lead by incorporating immunization equity indicators into performance metrics for health systems and provider networks.

Value-based payment models can reward providers who reduce disparities in vaccination coverage across different geographic or demographic groups. Programs such as New York’s Delivery System Reform Incentive Payment (DSRIP) initiative and Massachusetts Medicaid accountable care organizations illustrate how performance-linked financing can align delivery systems around measurable population outcomes (21, 22). While these programs vary in structure and outcomes, they demonstrate the feasibility of linking payment reform to preventive and equity-oriented objectives.

Extending preventive care incentives to commercial insurers could further reduce fragmentation between public and private payers. Aligning reimbursement structures with public health goals advances the RMIC principles of organizational and system integration, making equity a measurable performance expectation rather than a secondary objective.

#### 5.3.1 Implementation and governance considerations

In the United States, Medicaid programs are jointly financed by federal and state governments, with states retaining significant authority over benefit design and payment models under federal oversight. The Centers for Medicare & Medicaid Services (CMS) may approve state innovation through mechanisms such as Section 1115 demonstration waivers, which allow states to test alternative payment and delivery models within Medicaid programs (21).

Governance constraints include variation in state administrative capacity, differences in managed care penetration, and statutory limitations on performance-based reimbursement in certain jurisdictions. Resourcing implications include actuarial analysis, contract redesign, provider reporting infrastructure, and data integration capacity to measure immunization equity accurately.

Sequencing would likely involve (1) defining standardized immunization equity indicators, (2) incorporating them into Medicaid managed care quality benchmarks, (3) piloting performance-linked incentives in selected regions, and (4) evaluating effects before broader adoption. Collaboration between public health agencies and Medicaid authorities is essential to ensure that registry data and payer data are aligned.

Political and operational risks include provider resistance to additional reporting requirements, concerns about administrative burden, and unintended penalization of safety-net providers serving high-risk populations. Mitigation strategies include risk adjustment, phased implementation, technical assistance, and ensuring that incentive structures reward improvement as well as absolute performance.

### 5.4 Fund community partnered vaccine outreach

Building trust in immunization requires sustained collaboration between public health systems and community-based organizations. Partnerships with faith leaders, tribal councils, neighborhood groups, and other trusted entities can create culturally responsive outreach models that reflect community values and priorities.

Evidence from behavioral and public health research suggests that trusted messengers, repeated engagement, and locally tailored communication strategies are associated with improved vaccine confidence and uptake, particularly when structural barriers such as transportation, clinic hours, and language access are addressed (14–17). Community-partnered models therefore represent an important component of normative and organizational integration within the RMIC framework.

Dedicated funding should support these partnerships over time, enabling continuous engagement and capacity building. Mobile vaccination clinics can be integrated into these efforts so that families receive both education and services in familiar and trusted environments such as schools, community centers, or places of worship.

#### 5.4.1 Implementation and governance considerations

Authority for funding community-based outreach typically resides at the state and local public health level, often supported by federal immunization grant programs administered through agencies such as the CDC. Sustainable implementation therefore depends on coordination between federal funding mechanisms and state-level public health strategy.

Governance considerations include transparent allocation criteria, equitable distribution of funds across communities, and formal partnership agreements that clarify roles, expectations, and evaluation metrics. Contracts should prioritize capacity building and long-term relationship development rather than short-term campaign deliverables.

Resourcing implications include multiyear funding commitments, workforce training, translation and interpretation services, logistical support for mobile or school-based clinics, and administrative oversight to ensure continuity. Sequencing would likely involve (1) mapping under-immunized communities using registry data, (2) establishing partnership agreements with trusted local organizations, (3) co-designing outreach and service delivery strategies, and (4) evaluating uptake patterns and community feedback before scaling.

Political and operational risks include perceptions of politicized messaging, inconsistent funding continuity, and potential overreliance on unpaid community labor. Mitigation strategies include compensating community partners fairly, maintaining nonpartisan framing, embedding transparent evaluation mechanisms, and ensuring that outreach is paired with accessible service delivery to avoid placing responsibility solely on families to navigate complex systems.

Importantly, community partnership strategies should be integrated with the data infrastructure and policy alignment reforms described in prior sections. Normative integration strengthens trust, but it cannot substitute for structural investment in governance and delivery systems.

### 5.5 Incorporate pediatric immunization equity into national public health dashboards

Transparent and timely reporting is essential for accountability in population health. While national agencies report immunization trends, these data are often retrospective and not consistently integrated into routine decision-making processes.

Equity-focused dashboards should provide timely, disaggregated data by geography, race or ethnicity, socioeconomic status, and school district where feasible and legally permissible. Linking dashboards to immunization information systems and payer data can support more dynamic monitoring of coverage trends and disparities (12, 13). Publicly accessible reporting may also strengthen trust by making performance visible and understandable.

Dashboards alone, however, do not produce improvement. Their value depends on clearly defined accountability mechanisms and structured response pathways when performance declines.

#### 5.5.1 Implementation and governance considerations

Authority for national-level immunization reporting in the United States resides primarily with federal public health agencies, while data generation and registry maintenance remain largely state-based. Effective dashboard implementation would therefore require standardized reporting definitions, interoperability alignment, and cooperative federal-state agreements.

Governance considerations include compliance with privacy protections, appropriate aggregation thresholds to avoid re-identification in small communities, and safeguards against stigmatization of underperforming districts. Equity-disaggregated reporting must be paired with contextual interpretation to avoid attributing disparities to communities rather than to structural conditions.

Resourcing implications include investment in analytic infrastructure, data validation processes, and personnel trained in equity-focused data interpretation. Sequencing would likely involve (1) defining a core national immunization equity indicator set, (2) piloting dashboards in selected regions, (3) establishing review cadence and response protocols, and (4) scaling implementation based on operational lessons.

Accountability mechanisms should include predefined review intervals (e.g., annual national reporting with quarterly regional monitoring), publicly stated performance benchmarks, and structured decision points. When coverage in defined populations falls below agreed thresholds, response pathways may include targeted technical assistance, temporary funding adjustments, or intensified outreach support rather than punitive sanctions.

Political and operational risks include misinterpretation of data, politicization of performance comparisons, and potential penalization of under-resourced communities. Mitigation strategies include risk-adjusted comparisons, transparent methodology disclosure, and coupling performance reporting with capacity-building support.

When embedded within broader integration reforms, dashboards can shift immunization monitoring from retrospective reporting toward continuous performance management aligned with equity and governance accountability (Table 3).

**Table 3:** Rainbow model domains applied to pediatric immunizations.

RMIC domain	Description	Pediatric immunization example
Clinical integration	Coordination of preventive and clinical services across the care continuum	Electronic record prompts during well child visits and standardized workflows for catch up vaccinations
Professional integration	Collaboration and shared practices across disciplines and roles	Pediatricians, school nurses, pharmacists, and community health workers using common protocols and engaging in joint training
Organizational integration	Alignment and cooperation across institutions and service delivery organizations	Health departments, schools, clinics, and hospitals organizing coordinated back to school vaccination efforts
System integration	Policy, regulatory, and financing structures that support aligned actions	Harmonized immunization requirements, consistent reimbursement for vaccine delivery, and coordinated national to regional governance
Functional integration	Infrastructure that enables data exchange, communication, and operational support	Interoperable immunization information systems that provide real time data for clinics, schools, and public health agencies
Normative integration	Shared values, trust, and collective commitment across the system	Engagement of trusted community leaders to support culturally responsive outreach and strengthen vaccine confidence

### 5.6 Operationalizing integration and accountability

Translating the Rainbow Model of Integrated Care into implementation requires linking each integration domain to measurable system actions and predefined accountability processes, a challenge frequently identified in population health management implementation literature (23). A simplified logic sequence for pediatric immunization systems begins with governance and policy alignment, proceeds through interoperable data infrastructure and coordinated delivery, incorporates targeted community engagement, and culminates in continuous equity-focused monitoring with iterative adjustment.

To move beyond descriptive alignment, each RMIC domain may be associated with specific performance indicators and review mechanisms (Table 4). These indicators are illustrative rather than exhaustive and are intended to guide structured implementation rather than impose uniform metrics across jurisdictions.

**Table 4:** Illustrative indicators and accountability mechanisms by RMIC domain.

RMIC domain	Example measurable indicators	Accountability mechanisms
Clinical integration	% of well-child visits with documented immunization reconciliation; missed opportunity rate for vaccination	Quarterly clinic-level review; quality improvement cycles
Professional integration	Presence of cross-sector immunization collaboratives; participation in joint QI initiatives	Annual reporting of collaborative activity; interprofessional review meetings
Organizational integration	Formal coordination agreements between health departments and schools; jointly implemented campaigns	Regional performance review; shared accountability agreements
System integration	Alignment of exemption policy with baseline standards; payer contracts including immunization equity indicators	Legislative review cycles; payer performance audits
Functional integration	% of providers connected to interoperable IIS; timeliness of registry reporting	State registry audits; federal grant compliance benchmarks
Normative integration	Community vaccine confidence survey scores; community representation in planning bodies	Public reporting; community advisory oversight

Accountability requires predefined review cadence and response thresholds. Annual public reporting, supplemented by periodic regional monitoring, can support early identification of declining coverage or widening disparities. When performance falls below established benchmarks, response pathways—such as targeted technical assistance, resource reallocation, or policy review—should be triggered. Indicators must be interpreted within context and risk-adjusted where appropriate to avoid penalizing safety-net providers or under-resourced communities.

By pairing integration domains with measurable indicators and explicit review mechanisms, the Rainbow Model functions not only as a descriptive framework but also as an operational guide for governance and performance management.

## 6 Discussion

The persistent decline in pediatric immunization coverage reflects a deeper and longstanding challenge: the gap between population health theory and its practical implementation. Although the framework was designed to emphasize outcomes, social determinants, and equity, it has often been diluted in real-world practice. This conceptual drift, amplified by fragmented governance, inconsistent incentives, and competing interests, has hindered progress, particularly in decentralized and politicized systems such as the United States.

Wide variation in state-level policies and resource allocation further reinforces this drift. Differences in public health investment, preventive services, and social supports have deepened disparities and weakened the coherence of national health strategies, underscoring the need for alignment across federal and state systems (24).

A central obstacle lies in the tendency of organizations to reduce population health to panel management and cost containment rather than community engagement and equity. This narrow focus elevates organizational metrics while diminishing attention to clinical coordination, public trust, and patient experience. Normative dimensions such as shared values, power sharing, and cultural alignment are often treated as secondary, yet they are essential for public acceptance and sustained participation.

International experience further illustrates how durable integration structures can sustain high pediatric immunization coverage. Hungary provides a long-standing example through its state-funded health visitor network (védőnő), a nationally coordinated system of trained nurses responsible for preventive maternal and child health services, including immunization support. This model embeds clinical, professional, and organizational integration within a unified public health infrastructure, ensuring continuity from infancy through school age. Consistent with this integrated approach, Hungary has maintained near-universal childhood vaccination coverage, with measles immunization remaining near 100% of the target population in recent years. Such examples underscore that high performance is not solely a function of public trust, but of system design that aligns governance, workforce roles, and preventive delivery across sectors (25, 26).

Efforts to strengthen integration and accountability in immunization systems must also account for potential unintended consequences. Policies that link funding to performance, tighten exemption standards, or publicly report disaggregated coverage data carry ethical and operational trade-offs. Without careful design, performance-based incentives may disproportionately penalize under-resourced communities or safety-net providers that serve populations facing structural barriers to care. Similarly, enforcement mechanisms tied to school entry requirements may unintentionally burden families with limited access to transportation, flexible work schedules, or primary care continuity.

Data transparency initiatives, while essential for accountability, must also safeguard against stigmatization of communities with lower coverage. Disaggregated reporting can illuminate inequities, but if presented without contextual interpretation it may reinforce narratives that attribute disparities to community behavior rather than to structural constraints. Privacy protections and aggregation thresholds are necessary to prevent re-identification in small populations and to maintain trust in immunization information systems.

To mitigate these risks, integration strategies should incorporate equity safeguards at each stage of implementation. Performance benchmarks can emphasize improvement over absolute thresholds, funding adjustments can be paired with technical assistance rather than punitive withdrawal, and exemption policy reform can include medical vulnerability protections and due process safeguards. Community representation in governance structures further strengthens accountability by ensuring that policy responses are informed by lived experience rather than imposed externally.

Recognizing these trade-offs does not weaken the case for integration. Rather, it underscores the importance of coupling structural reform with ethical vigilance and participatory governance. Population health frameworks achieve legitimacy not only through measurable outcomes but also through fairness in design and implementation.

The drift extends beyond implementation to the meaning of the framework itself. Although population health language is now common in policy and institutional planning, initiatives often prioritize performance indicators while overlooking the structural determinants that shape inequity (27). Reclaiming the framework’s equity focus requires operational clarity and accountability at every level of governance.

Addressing these normative and structural gaps demands a shift from race-based risk framing toward a structural understanding of inequity. Disparities arise not from racial identity, but from cumulative exposure to unequal policies, institutional practices, and resource distributions across healthcare, housing, education, and employment. Accountability must therefore extend to the systems that create and maintain these conditions (5, 21, 28). Without this perspective, even well-intentioned interventions risk reinforcing existing disparities.

Pediatric immunizations illustrate that alignment on theory is not sufficient. Progress depends on operational coherence, community informed design, and infrastructure capable of supporting coordinated and equitable responses. Encouragingly, several initiatives demonstrate that population health principles can be applied effectively. The Camden Coalition model shows how data sharing and person centered engagement can improve outcomes for patients with complex needs (22, 29). Kaiser Permanente’s Thrive initiative integrates electronic records, education, and community partnerships to support prevention (30). New York’s Delivery System Reform Incentive Payment program links financial incentives to Medicaid performance and equity (31), while Massachusetts Medicaid accountable care organizations integrate social determinant screening with community partnerships to address upstream barriers (32) (Table 5).

**Table 5:** Common elements of successful population health framework implementations.

Program or model	Core features	Aligned RMIC domains
Camden coalition (Hotspotting)	Cross sector data integration, intensive care coordination, and engagement of individuals with complex medical and social needs	Functional, clinical, organizational
Kaiser permanente thrive initiative	Electronic record based outreach, emphasis on prevention and wellness, and strong partnerships with community organizations	Clinical, professional, normative
New York State DSRIP program	Performance based financial incentives, regional provider networks, and coordinated strategies to improve outcomes for publicly insured populations	Organizational, system, functional
Massachusetts medicaid accountable care organizations	Screening for social determinants of health, integration of community based organizations, and shared savings models to support care redesign	Clinical, organizational, normative
Pediatric immunization delivery (United States)	Fragmented data systems, inconsistent mandates, variable outreach capacity, and uneven collaboration across sectors	All RMIC domains with particular gaps in system, normative, and functional integration

Despite their differences, these programs share common features: cross sector collaboration, data infrastructure, localized governance, and clear accountability. The Rainbow Model of Integrated Care provides a framework for organizing these features and diagnosing where coordination breaks down. Unlike broad conceptual descriptions, the model translates population health into actionable domains that encompass technical systems, professional collaboration, organizational alignment, and community trust. In pediatric immunization, where several domains remain underdeveloped, the model clarifies where structural investment and governance reform are most needed.

Advancing population health requires moving from fragmented, program specific efforts to integrated systems that promote collaboration, accountability, and equity (33). Sustained progress depends on treating health as a collective societal responsibility supported by shared governance and long-term investment across sectors.

Population health frameworks are most likely to achieve their intended impact when equity becomes a core metric of accountability. Embedding structured models such as the Rainbow Model and centering community voice in both design and delivery can shift population health from aspiration to system condition. Pediatric immunizations offer a clear and measurable test case. If the framework struggles in domains where evidence is strong and alignment is achievable, it signals deeper challenges in how health systems govern for equity (33).

### 6.1 Limitations

This analysis has several limitations. First, it is informed by a structured narrative review rather than a systematic evidence synthesis. Although sources were selected to reflect major empirical findings, policy developments, and implementation frameworks, selection was non-systematic and may not capture all relevant interventions or contradictory findings. No formal grading of evidence strength was conducted. Second, while pediatric immunizations provide a clear and measurable case example, the analysis is grounded primarily in the United States policy environment. Although comparative reference is made to international systems, legal authority, financing mechanisms, and governance structures vary across countries. The feasibility and sequencing considerations discussed here may therefore require adaptation in other contexts. Third, the manuscript emphasizes integration and governance design but does not include formal cost-effectiveness modeling, detailed fiscal projections, or comprehensive legal analysis of constitutional constraints. Implementation capacity also varies widely across jurisdictions, and resource limitations may affect the pace and scope of reform. Finally, although measurable indicators are proposed to operationalize integration, these are illustrative rather than prescriptive. Further empirical research is needed to validate which combinations of policy alignment, infrastructure investment, and community engagement strategies most effectively improve equitable immunization coverage.

Acknowledging these limitations underscores that the Rainbow Model of Integrated Care is presented as an organizing framework for policy analysis rather than as a definitive or exhaustive solution.

## 7 Conclusion

Pediatric immunizations serve as both a measure of system performance and a practical test of how population health principles are translated into implementation. Declining coverage and widening geographic disparities highlight the limitations of relying on broad conceptual frameworks without corresponding operational structure, governance alignment, and accountability mechanisms.

The Rainbow Model of Integrated Care offers a structured lens through which fragmentation can be identified, and integration can be intentionally designed. By linking clinical coordination, professional collaboration, organizational alignment, policy design, functional infrastructure, and normative trust, the model clarifies where structural gaps persist and where targeted reform may be most effective. Importantly, integration must be paired with measurable indicators, transparent review processes, and equity safeguards to avoid unintended consequences.

This analysis illustrates how structured integration frameworks can inform policy design and implementation planning in immunization systems. It does not suggest that a single model will eliminate variation or political disagreement, nor that structural reform alone guarantees improved uptake. Rather, it argues that clearer governance pathways, interoperable infrastructure, aligned incentives, and participatory accountability mechanisms are necessary conditions for sustainable progress.

Advancing population health requires moving beyond aspirational language toward coordinated and ethically grounded system design. Pediatric immunizations provide a visible and measurable starting point. If integration can be operationalized in this domain—where evidence is strong and prevention is well established—it may offer a template for strengthening equity and coherence across broader health system challenges.
